# Nutritional Characteristics of Black Lentil from Soleto: A Single-Flower Vetch Landrace of Apulia Region (Southern Italy)

**DOI:** 10.3390/foods10112863

**Published:** 2021-11-18

**Authors:** Angela Rosa Piergiovanni

**Affiliations:** Institute of Biosciences and Bioresources, National Research Council, Via G. Amendola 165/a, 70126 Bari, Italy; angelarosa.piergiovanni@ibbr.cnr.it

**Keywords:** culinary traits, seed composition, total phenolic compounds, *Vicia articulata* Hornem

## Abstract

Archaeological remains and historical documents demonstrate that a single-flower vetch has been cultivated in Italy from the early stages of agriculture. Some Italian communities have perpetuated the custom to eat its seeds still to the present. This is the case of people living in some villages of the southern Apulia region. In consequence of the high resemblance of the single-flower vetch (*Vicia articulata* Hornem.) seeds with those of lentils, the Apulian landrace is locally named “*lenticchia nera di Soleto*” (black lentil from Soleto). The evaluation of seed nutritional traits of this landrace revealed good macronutrient contents (proteins and starch, 28.4 and 42.4 g/100 g respectively), low trypsin inhibitor levels (4.08 TIU/mg), short cooking times after soaking (24–25 min) and a lack of broken seeds at the end of cooking. The coat content of total phenolic compounds (TPC) of the Apulian black lentil was comparable with that of the lentil *cv.* Beluga (68.23 vs. 66.14 mg GAE/g, respectively).

## 1. Introduction

Legumes have had a great relevance throughout human communities from the early stages of agriculture. Over time, several annual and perennial species were domesticated in both the Old and New World [1]. Those with good agronomic and nutritional performances are still cultivated and eaten at a worldwide level. The success of legumes is attributable to their many uses (food, feed, foraging, green manure, and ornamental and medicinal utilization), their easy seed storage, and the multiple ways of their consumption (leafs, immature pods and seeds, dry seeds). Recently, frozen immature pods and seeds have also gained consumer approval.

The genus *Vicia* comprises more than 160 species mainly diffused in Mediterranean type climates. Nowadays, only a few annual species, such as the fava bean (*V. faba* L.), common vetch (*V. sativa* L.), broad-leaved vetch (*V. narbonensis* L.), hairy vetch (*V. villosa* Roth), bitter vetch (*V. ervilia* L.) and Hungarian vetch (*V*. *pannonica* L.), have relevance from an economical point of view. The fava bean is a staple food in several countries, while the other listed species are appreciated for feed or foraging with good performances in areas characterized by cold winters or dry conditions. In the past, in temperate areas of the northern hemisphere, some vetches were usually included in human and/or animal alimentation [2,3,4]. Archaeological findings proved that, in addition to cereals, peas, lentils, *Lathyrus* spp. and vetches were among the Near Eastern founder crops [5]. In Europe, they were part of the daily diet of hunter-gatherers at the end of the last Ice Age [6]. The cultivation began during the 10th and 9th millennia BC in the Near East Mediterranean regions, as testified by the vetch presence in the remains of seed mixtures [3,5]. The legume’s importance as major crops declined with animal domestication and pastoralist diffusion. The achievement of a constant supply of milk and meat, and therefore of proteins, reduced the importance of some species as staple foods. The vetch species experienced a dramatic decrease in cultivation, mainly attributable to a lower nutritional value with respect to other legumes [7,8,9]. Despite this, the custom to eat vetch seeds has sporadically survived to the present in a few Mediterranean countries such as Italy [10,11,12].

The Italian custom of including some vetches in the diet is testified by short mentions in some books published over the past two centuries. This is the case of the black lentil (*Vicia articulata* Hornem.) in Apulia [13,14,15], Sicily [16] and Sardinia, and the “*mavrifacì*” (bitter vetch) in Calabria (http://ilgiardinodellemeraviglie.info/bioarchivio/lenticchia-nera-di-bova-o-mavrafaci/ (accessed on 16 June 2021)). The vetch most commonly consumed in some Italian areas is the single-flower vetch, whose seeds closely resemble those of the lentil to the point that it is commonly named “*lenticchia nera*” (black lentil). Although it did not have a pleasant taste, farmers appreciated this vetch for the higher yield and pest resistance with respect to the lentil. Indeed, the number of seeds per pod ranges from three to five in contrast with one to two seeds typical of the true lentil. Maly et al. [16] detected relic cultivations at Gallodora and Mongiuffi (two Sicilian villages) and referred to the custom of inhabitants to eat its seeds. The vanishing of the same habit at Salina, a small island to the north-west of Sicily, has been recently reported [17]. The cultivation and consumption of single-flower vetch survives at Calasetta, a village of S. Antioco, a small island to the south-west of Sardinia [18] and Corigliano d’Otranto, Soleto and Martano, three villages of southern Apulia [19]. These landraces are locally named “*lentiggia naigra de Cadesedda*” (black lentil from Calasetta) and “*lenticchia nera from Soleto”* (black lentil from Soleto), respectively. Unfortunately, only fragmentary information about the nutritional traits of these landraces is available in the literature (Laghetti et al., 2000). The purpose of this paper was to try to fill this gap: (1) by investigating in detail the nutritional traits of the Apulian “*lenticchia nera*”; and (2) by comparing the results with those of the “*lentil from Altamura*”, an appreciated Apulian lentil landrace [20] that recently obtained the Protected Geographic Origin (PGI) mark, and the black lentil cultivar Beluga.

## 2. Materials and Methods

### 2.1. Plant Materials

About 200 g of dry seeds of the landraces “*lenticchia nera from Soleto”* (*Vicia articulata* Hornem.) and “*lentil from Altamura”* (*Lens culinaris* Medik.) were obtained from two Apulian farmers, who annually grow them. The landraces were cultivated during the growing season 2016–2017 in their respective traditional environment, according to the traditional agro-techniques. The fields were located in the countryside of Corigliano d’Otranto (lat 40.1165, lon 18.2514, elevation 79 m a.s.l.) and Altamura (lat 40.8564, lon 16.6001, elevation 433 m a.s.l.). The sowing of the landraces occurred in December and the harvest in June. The *cv*. Beluga (*Lens culinaris* Medik.) was purchased in a market.

### 2.2. Seed Composition

About 80 g of dry seeds of the two landraces were ground in a Cyclotec 1093 laboratory mill (Tecator, Sweden) to obtain a fine meal used for chemical analyses. Moisture was quantified by weight loss according to the AOAC method 930.15 [21]. Seed protein content was measured by the micro-Kjeldahl method (N × 6.25) according to the AOAC methodology 979.09 [21]. Ash content was determined gravimetrically according to the AOAC method 923.03 [21]. Total starch was evaluated according to Garcia-Alonso et al. [22]. Briefly sampled meal was suspended in KOH 2M (8:1, *w*/*v*), treated with amyloglucosidase and measured as glucose. Starch was equal to glucose × 0.9. Total phenolic compounds (TPCs) were measured on whole seeds, and on coats and cotyledons separately using the Folin–Ciocalteau colorimetric assay as modified by Lin and Lai [23]. Briefly, TPCs were extracted at 40 °C for 1 h with a mixture of MeOH:H_2_O (3:2, *v*/*v*) acidified with HCl (0.1%). Extraction was repeated two times and supernatants were pooled before the analysis. Diluted Folin–Ciocalteau reagent was added to extract aliquots and the mixture was neutralized with sodium carbonate. Absorbance was measured at 760 nm. TPCs content was expressed as the gallic acid equivalent (GAE). Cooked seeds were lyophilized before the extraction of TPCs. Trypsin inhibitors (TI) were spectro-photometrically determined by extracting defatted meals with a glycine buffer (pH 11). The assay was carried out as previously described [24]. The TI content was expressed as a unit of inhibitors per miligram of dry matter.

All used enzymes were purchased from Sigma-Aldrich (St. Louis, MO, USA), and the chemicals were reagent grade.

### 2.3. Culinary Traits

Fifty seeds, randomly selected, were used for the measurement of coat percentage. Seeds were soaked overnight in tap water at room temperature. Coats, manually separated from cotyledons, were lyophilized and weighted. Coat percentage was quantified with respect to whole seed weight. Randomly selected seeds, one hundred for each test, were used for the determination of the hydration index (HI) and swelling index (SI). HI at time *t* was equal to the ratio of the seed weight increase after soaking and the weight before soaking. SI at time *t* was the ratio of the seed volume increase after soaking and the seed volume before soaking [25]. These values were expressed as a percentage. Six grams of seeds were used for cooking time measurements. The test was carried out in an open vessel heated with an electric burner to mimic usual domestic cooking. After 20 min of heating, the softness of ten seeds was checked every 2 min until cooking was completed. The cooking was considered complete when, after pressing the seeds between two glass slides, no uncooked parts remained. Single seed weight after cooking was measured on twenty integer seeds blotted to remove all cooking water. Seeds of the black lentil were soaked overnight before cooking, while the *lentil from Altamura* and cv Beluga did not require being hydrated before the test. The experimental data were reported as mean ± SD, mean values of recorded traits were subjected to Duncan’s test. The Statistica software package, version 7.1 (StatSoft, Tulsa, OK, USA) was used.

## 3. Results and Discussion

### 3.1. Seed Morphology

**Figure 1:**
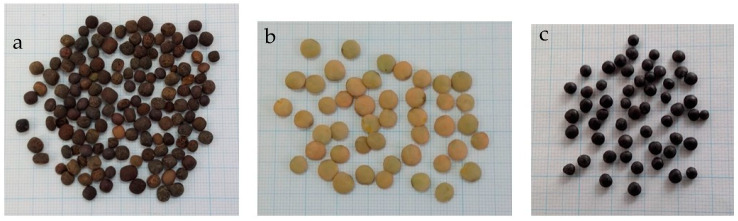
Seeds of tested landraces. *lenticchia nera from Soleto* (**a**); *lentil from Altamura* (**b**); *cv*. Beluga *(***c**).

A visual inspection of the “*lenticchia nera from Soleto*” seeds allowed recognition of morphological differences with respect to the two lentils included in the study (Figure 1). “*Lenticchia nera from Soleto*” seeds were round in shape, with a mean diameter of 3 mm, but they were not flat as those of lentils usually are. The seed thickness was about 2 mm. Despite the name, the coat color of Apulian landrace was not black, such as the *cv.* Beluga. The background coat color was dark brown and the coat was marbled; the cotyledon color was greenish-yellow or orange.

The seed traits of the “*lenticchia nera from Soleto*” highly resembled those of the single-flower vetch grown at Calasetta, S. Antioco Island, Sardinia [18]. It is worthy noticing that the local name of this landrace is also the black lentil (*“lentiggia naigra”),* suggesting that the confusion between the single-flower vetch and the lentil has been, and still is, recurrent among Italian farmers. The weight of 1000 seeds resulted higher for the Apulian landrace with respect to the Sardinian one (63.8 g vs. 55 g, respectively) [18]. However, it should be taken into consideration that the values refer to seed batches grown in different years and environments.

### 3.2. Seed Composition

Seed composition of the *“lenticchia nera from Soleto”* and the “*lentil from Altamura”*, harvested in the 2016–2017 growing season, as well as that of the *cv. Beluga*, are summarized in Table 1. Overall, the *“lenticchia nera from Soleto”* showed good macronutrient (proteins and starch) contents, a low trypsin inhibitor level and a short cooking time after soaking. This could justify its inclusion in the human diet until the XX century by the poorest Apulian people, not only in periods of food shortage.

In other legume species [26], starch resulted as the major macronutrient followed by proteins (42.4 and 28.4%, respectively). However, this starch content was significantly inferior (*p* ≤ 0.01) to the values shown by the two lentils (Table 1), as well as to the values reported in the literature for other lentils [22,26]. Conversely, the Apulian vetch was significantly higher in proteins with respect to the *lentil from Altamura* and *cv.* Beluga (24.4 and 25.0%, respectively), suggesting a good nutritional value of this landrace. Moreover, the protein content of the *“lenticchia nera from Soleto”* was also significantly higher than the value (23.6%) reported for the Sardinian black lentil [18]. This disagreement can be attributed to the comparison of seed batches cultivated in different growing seasons and environments. When the protein value recorded in this study for the *“lenticchia nera from Soleto*” was compared with those available in the literature, it resulted within the range (22–31%) reported for the Spanish germplasm collection of the single-flower vetch [27].

Of course, a correct comparison of nutritional value associated with different species should take into consideration not only protein content but also the presence of species-specific protein fractions. These fractions, if present, could have adverse effects on amino acid composition, nutritional value and digestibility. Indeed, this was observed by comparing the profile of alcoholic/saline extracts obtained from lentils and some vetch species [28]. Authors reported that some protein fractions highly expressed in the single-flower vetch profiles were completely absent in the lentil. Pastor-Cavada et al. [29], who investigated the amino acid composition in 28 *Vicia* spp., found an affinity of 94.6% among the single-flower vetch, fava bean and four other annual vetches. All these species were characterized by higher contents of leucine and lower amounts of valine with respect to the remaining ones. Like in lentils [30], and in general legume species, glutamic acid was predominant in the amino acid profile of the single flower vetch while the lowest levels were associated with sulphur-containing amino acids.

As proved by several studies, the several classes of antinutritionals present in legume seeds have negative effects on their nutritional value and palatability; nevertheless, some of them also have healthy properties. For example, trypsin inhibitors (TIs) reduce digestion and absorption of dietary proteins from human consumers and monogastric animals, but it has been demonstrated that they have anti-carcinogenic and anti-inflammatory activity [31]. The TI level significantly differs among legume species and, within each species, among cultivars, landraces and populations [32]. Previous studies showed that the lentil, fava bean and some *Vicia* species have TI contents lower than those of widely consumed legumes such as the common bean and soybean [7,33]. In agreement with the literature, the “*lenticchia nera from Soleto*” showed a low TI content and did not significantly differ from the values of the two lentils included in this study (Table 1). It is worth underlining that TIs, being thermolabile compounds, lose a great part of their activity during cooking. Consequently, they have a negligible effect on digestion and absorption of the “*lenticchia nera from Soleto*” proteins by human consumers. On the other hand, the low TI content of this landrace makes its seeds safer, in the appropriate amount, than other legumes in feed for monogastric animals. For example, for some cultivars of the soybean and chickling vetch, usually used as feed, higher TI content (55.9–62.6 and 21.6–29.0 TIU/mg, respectively) has been reported in the literature [34].

### 3.3. Total Phenolic Componds

Phenolic compounds are secondary metabolites widespread in wild and edible plants. They play several roles, from the defence against pests to plant growth regulators. The interest of nutritionists toward total phenolic compounds (TPCs) comes from the health benefits associated with them. The several classes of these compounds (phenolic acids, flavonoids and condensed tannins), commonly named TPCs, have antioxidant, antimicrobial, and anti-inflammatory properties, and might offer protection against some degenerative diseases [35]. The incorporation of phenolic extracts in some food and cosmetic formulations has been recently exploited to take advantages from these properties [36,37]. Studies aimed to quantify TPCs content in the legume species showed that the lentil is characterized by higher levels with respect to the soybean, chickpea and pea [26,38]. In legumes, TPCs are concentrated in the seed coats, so within each species, pigmented seeds have higher contents with respect to pale colored or white coat counterparts [37]. As expected, due to the dark coat colour, whole seeds of the “*lenticchia nera from Soleto*” had a significantly (*p* ≤ 0.01) higher TPCs content than that of the *lentil from Altamura* (Table 1), the coat of which is a shade of brown considerably more pale (Figure 1). However, the value was significantly lower (*p* ≤ 0.01) than that recorded for the *cv.* Beluga (6.02 vs. 10.87 mg GAE/g_ss_.) the seed coat of which is black (Figure 1). Although, data on TPCs content in the single-flower vetch available in the literature are very scarce and the comparison of data reported by different authors is difficult, due to the use of different methods of analysis, the value of the present study was comparable with that reported by Berger et al. [7] for the common vetch. The distribution of TPCs between the coat and cotyledon was also investigated. As expected, TPCs were mainly present in the coats of the three compared samples (Table 1), but the calculation of the ratio TPCs coat/TPCs cotyledon highlighted differences among them. This ratio was 30.3 for the “*lenticchia nera from Soleto*”, 58.4 for the *lentil from Altamura* and 32.7 for the *cv.* Beluga. A detailed analysis of TPC values relative to the two seed components showed an evident dissimilarity of the *lentil from Altamura.* It had a remarkably higher content for the coat and a much lower cotyledon value than those recorded for the “*lenticchia nera from Soleto*” and *cv.* Beluga (Table 1). Although further investigations are required to explain these results, it was undoubted that the “*lenticchia nera from Soleto*” showed a greater similarity with the *cv.* Beluga for TPC distribution in seed tissues.

It is widely known that cooking produces significant changes in bioactive compounds present in legume seeds, and that TPC content decreases during soaking and cooking [38]. If a soaking period is required before cooking the “*lenticchia nera from Soleto*”, seeds of the *lentil from Altamura* and the *cv.* Beluga, similar to almost all lentils, they did not need to be rehydrated. As shown in Table 1, the TPC level drastically decreased after the different processes undergone by seeds (soaking and cooking or only cooking) and only a residual quantity remained in cooked seeds. The loss exceeded three quarters of the initial values, being 74.1, 79.9 and 82.9% for the *lentil from Altamura,* the “*lenticchia nera from Soleto*” and the *cv.* Beluga, respectively. These results show that, as for lentils, the potentially high bioactivity of TPCs present in crude extracts of the single-flower vetch is remarkably lost during the seed processing required for their consumption.

### 3.4. Culinary Traits

**Figure 2:**
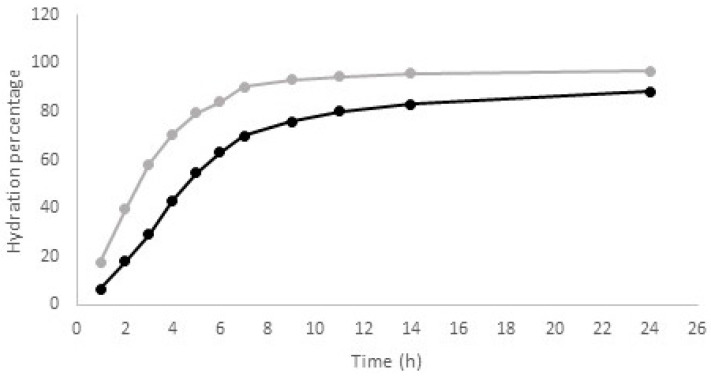
Water absorption curve recorded for the “*lenticchia nera from Soleto*” and the *cv.* Beluga (grey and black trace, respectively).

Seeds of the “*lenticchia nera from Soleto*” needed to be soaked before cooking. As seed hydration is a time consuming step, the knowledge of the optimal soaking time for each species and within each one for each landrace or cultivar, has great relevance for consumers. It is known that seed hydration is not a simple diffusional process of water across the coat and cotyledon. Two different models, the downward concave shape (DSC) and the sigmoidal shape, describe the hydration kinetic of legume species [39]. The first one can be applied to seeds with a permeable coat, while the second one is required to describe seed hydration that occurs through the hilum. Due to the lack of information about the hydration kinetics of the single-flower vetch seeds, this parameter was also investigated. Experimental results showed that for the “*lenticchia nera from Soleto*”, the water uptake occurred in agreement with the DSC model (Figure 2), the same description of seed hydration as the lentil. These preliminary data suggest that, similarly to the lentil, the coat of this Apulian landrace should be permeable to water.

From a nutritional point of view, the cooking of legume seeds is a necessary stage to gelatinize starch, increase protein digestibility, and inactivate the great part of antinutritional compounds. For practical reasons, legume cooking time matters to consumers who are interested in time-saving during home-preparation of legume dishes. Generally within each legume species, varieties of landraces characterized by short cooking times are preferred. As shown in Table 1, the three tested samples were cooked in less than 36 min. As predicted, the shortest cooking time (24–25 min) was recorded for the pre-soaked seeds of the “*lenticchia nera from Soleto*”. This value was comparable with that of the *cv.* Beluga (26–27 min), of which the seeds were not soaked but were a very small size. A short cooking time is an incontestable advantage, as the loss of nutrients in the cooking water is proportional to the cooking duration [40]. The cooking yield was also high (Table 1). The average weight of the single cooked seed was doubled with respect to the initial weight of the *lentil from Altamura* and the *cv.* Beluga, both not soaked, while the “*lenticchia nera from Soleto*” was 130% with respect to dry single seed weight. Finally, the visual aspect of the cooked seeds revealed the lack of broken seeds for the “*lenticchia nera from Soleto*” and the *cv.* Beluga, while about 10% of the seeds of the *lentil from Altamura* lost their coats during cooking.

## 4. Conclusions

The Apulian landrace, named the “*lenticchia nera from Soleto*”, is part of the plant genetic heritage as well as of the traditional regional gastronomy. Unfortunately, it is in danger of disappearing in a very short time for several reasons, such as the general decline of legume consumption in Italy, and its frequent association to periods of food shortage. The presented results suggest reconsidering the nutritional reputation of this landrace and, more generally, of the single-flower vetch. Several seed traits showed values comparable with those of two lentils commonly bought by consumers. Moreover, the adaptation of this landrace to the Apulian climate is an important feature to be taken into consideration in future scenarios of environmental changes, as well as to promote the safeguard of agro-biodiversity. Local and national authorities should sustain the on-farm maintenance of landraces, which are bearers of useful nutritional characteristics.

## Figures and Tables

**Table 1:** Results of physico-chemical and nutritional seed analyses. All values are expressed on dry seed weight base.

Seed Trait	*lenticchia nera* (Black Lentil)	*lentil from Altamura*	*cv. Beluga*
1000 seed weight (g)	63.8 ± 0.219 a	59.1 ± 0.062 a	23.8 ± 1.09 b
Protein (g/100 g_ss_)	28.4 ± 0.312 a	24.4 ± 0.290 b	25.0 ± 0.252 b
Starch (g/100 g_ss_)	42.4 ± 1.041 a	50.2 ± 1.202 b	54.1 ± 1.604 b
Ash (g/100 g_ss_)	7.1 ± 0.081 a	6.5 ± 0.068 b	6.7 ± 0.092 b
TPC whole seeds (mg GAE/g_ss_)	6.02 ± 0.181 a	5.79 ± 0.103 b	10.87 ± 0.186 c
TPC cotyledons (mg GAE/g_ss_)	2.25 ± 0.111 a	1.52 ± 0.020 b	2.02 ± 0.024 c
TPC coats (mg GAE/g_ss_)	68.23 ± 0.570 a	88.70 ± 1.605 b	66.14 ± 1.990 a
TPC cooked seeds (mg GAE/g_ss_)	1.21 ± 0.052 a	1.50 ± 0.085 b	1.86 ± 0.037 c
Trypsin inhibitors (TIU/mg_ss_)	4.08 ± 0.192 a	3.71 ± 0.242 a	3.93 ± 0.190 a
Coat (g/100 g)	9.2 ± 0.046 a	6.9 ± 0.042 b	7.3 ± 0.050 c
Hydration index (% at 24 h)	88.0 ± 0.354 a	97.1 ± 0.177 b	96.7 ± 0.374 b
Swelling index (% at 24 h)	82.1 ± 0.551 a	110.0 ± 0.854 b	80 ± 0.950 a
Cooking time (min)	24–25 * a	33–35 ** b	25–26 ** a
Single seed weight before cooking (g)	0.120 ± 0.034 * a	0.062 ± 0.013 b	0.024 ± 0.006 c
Single seed weight after cooking (g)	0.15 ± 0.035 a	0.12 ± 0.018 a	0.052 ± 0.008 b

* after 16 h of soaking ** without soaking; TIU—trypsin inhibitor unit. Values followed by the same letter are not significantly different (*p* ≥ 0.01).

## Data Availability

Not applicable.

## References

[1] Fuller D.Q., Willcox G., Allaby R.G. (2011). Cultivation and domestication had multiple origins: Arguments against the core area hypothesis for the origins of agriculture in the Near East. World Archaeol..

[2] Valamoti S.M., Morel J.-P., Mercuri A.M. (2009). Plant food ingredients and ‘recipes’ from Prehistoric Greece: The archaeobotanical evidence. Plants and Culture: Seeds of the Cultural Heritage of Europe.

[3] Mikić A., Mihailović V., Ćupina B., Đorđević V., Milić D., Duc G., Stoddard F.L., Lejeune-Hénaut I., Marget P., Hanocq E. (2011). Achievements in breeding autumn-sown annual legumes for temperate regions with emphasis on the continental Balkans. Euphytica.

[4] Rubiales D., Mikić A. (2015). Introduction: Legumes in Sustainable Agriculture. Crit. Rev. Plant Sci..

[5] Mikić A. (2016). Presence of vetches (*Vicia* spp.) in agricultural and wild floras of ancient Europe. Genet. Resour. Crop. Evol..

[6] Aura J.E., Carrión Y., Estrelles E., Jordà G.P. (2005). Plant economy of hunter-gatherer groups at the end of the last Ice Age: Plant macroremains from the cave of Santa Maira (Alacant, Spain) ca. 12000–9000 b.p. Veg. Hist. Archaeobotany.

[7] Berger J., Robertson L., Cocks P. (2003). Agricultural potential of Mediterranean grain and forage legumes: 2) Anti-nutritional factor concentrations in the genus Vicia. Genet. Resour. Crop. Evol..

[8] Sadeghi G., Pourreza J., Samei A., Rahmani H. (2009). Chemical composition and some anti-nutrient content of raw and processed bitter vetch (*Vicia ervilia*) seed for use as feeding stuff in poultry diet. Trop. Anim. Health Prod..

[9] Uzun A., Güçer S., Açıkgöz E. (2011). Common Vetch (*Vicia sativa* L.) Germplasm: Correlations of Crude Protein and Mineral Content to Seed Traits. Plant Foods Hum. Nutr..

[10] Francis C.M., Enneking D., Abd El Moneim A.M., Knight R. (1999). When and where will vetches have an impact as grain legumes?. Linking Research and Marketing Opportunities for Pulses in the 21st Century, Proceedings of the Third International Food Legume Research Conference, Adelaide, Australia, 22–26 September 1997.

[11] Pinela J., Carvalho A.M., Ferreira I.C. (2017). Wild edible plants: Nutritional and toxicological characteristics, retrieval strategies and importance for today’s society. Food Chem. Toxicol..

[12] Peña-Chocarro L., Jordà G.P., Alonso N., Antolín F., Teira-Brión A., Tereso J.P., Moya E.M.M., Reyes D.L. (2019). Roman and medieval crops in the Iberian Peninsula: A first overview of seeds and fruits from archaeological sites. Quat. Int..

[13] Bruni A. (1845). Breve Ragguaglio Dell’agricoltura E Pastorizia Del Regno di Napoli.

[14] De Cesare C. (1859). Delle Condizioni Economiche E Morali Delle Classi Agricole Nelle Tre Provincie di Puglia.

[15] Castelli G. (1935). La coltivazione della lenticchia in provincia di Bari. La Propaganda Agricola XIII.

[16] Maly R., Hammer K., Lehamann C.O. (1987). Sammlung pflanzlicher genetischer Ressourcen in Sűditalien-ein Reisebericht aus dem Jahre 1950 mit Bemerkungen zum Schicksal der Landsorten “in situ" und in der Genbank. Kulturpflanze.

[17] Istituto Superiore per la Protezione e la Ricerca Ambientale (ISPRA) (2013). Frutti Dimenticati e Biodiversità Recuperate.

[18] Laghetti G., Piergiovanni A., Galasso I., Hammer K., Perrino P. (2000). Single-flowered vetch (*Vicia articulata* Hornem.): A relic crop in Italy. Genet. Resour. Crop. Evol..

[19] Accogli R., Dimitri G., Marchiori S. Lenticchia nera di Soleto: Storia locale di un legume minore. Proceedings of the IX Biodiversity National Congress.

[20] Piergiovanni A. (2000). The evolution of lentil (*Lens culinaris* Medik.) cultivation in Italy and its effects on the survival of autochthonous populations. Genet. Resour. Crop. Evol..

[21] AOAC, Association of Official Agricultural Chemists (1970). Official Methods of Analysis.

[22] García-Alonso A., Goñi I., Saura-Calixto F. (1998). Resistant starch and potential glycaemic index of raw and cooked legumes (lentils, chickpeas and beans). Z. Lebens Unters A.

[23] Lin P.-Y., Lai H.-M. (2006). Bioactive Compounds in Legumes and Their Germinated Products. J. Agric. Food Chem..

[24] Della Gatta C., Piergiovanni A.R., Perrino P. (1988). An improved method for the determination of trypsin inhibitor levels in legumes. Lebens-Wiss. Technol..

[25] Piergiovanni A., Cerbino D., Della Gatta C. (2000). Diversity in seed quality traits of common bean (*Phaseolus vulgaris* L.) populations from Basilicata (Southern Italy). Plant Breed..

[26] Costa G.E.D.A., Queiroz-Monici K.D.S., Reis S.M.P.M., de Oliveira A.C. (2006). Chemical composition, dietary fibre and resistant starch contents of raw and cooked pea, common bean, chickpea and lentil legumes. Food Chem..

[27] Vioque R.S., Prado I.C., Gil F.F., Alvear M.J.G., Pascual M.D.L.M., Conde M.F.R. (2008). Contents of total protein, L-canavanine and condensed tannins of the one-flowered vetch (*Vicia articulata* Hornem.) collection of the Bank of Plant Germplasm of Cuenca (Spain). Genet. Resour. Crop. Evol..

[28] Piergiovanni A.R., Taranto G. (2005). Simple and rapid method for the differentiation of *Lens culinaris* Medik. by false lentil species. J. Agric. Food Chem..

[29] Pastor-Cavada E., Juan R., Pastor J.E., Alaiz M., Vioque J. (2011). Nutritional Characteristics of Seed Proteins in 28 *Vicia* Species (*Fabaceae*) from Southern Spain. J. Food Sci..

[30] Khazaei H., Subedi M., Nickerson M., Martínez-Villaluenga C., Frias J., Vandenberg A. (2019). Seed Protein of Lentils: Current Status, Progress, and Food Applications. Foods.

[31] Piergiovanni A.R., Galasso I., Lioi L., Wilson D.G. (2017). Protease inhibitors in *Phaseolus* spp. Seeds. Seed Proteins. Biochemistry, Functional Properties and Health Benefits.

[32] Piergiovanni A.R., Pignone D. (2003). Effect of year-to-year variation and genotype on trypsin inhibitor level in common bean (*Phaseolus vulgaris* L.) seeds. J. Sci. Food Agric..

[33] Guillamón E., Pedrosa M.M., Burbano C., Cuadrado C., Sánchez M.D.C., Muzquiz M. (2008). The trypsin inhibitors present in seed of different grain legume species and cultivar. Food Chem..

[34] Pisulewska E., Pisulewski P. (2000). Trypsin inhibitor activity of legume seeds (peas, chickling vetch, lentils, and soya beans) as affected by the technique of harvest. Anim. Feed. Sci. Technol..

[35] Durazzo A., Lucarini M., Souto E.B., Cicala C., Caiazzo E., Izzo A.A., Novellino E., Santini A. (2019). Polyphenols: A concise overview on the chemistry, occurrence, and human health. Phytother. Res..

[36] Barroso M.R., Barros L., Dueñas M., Carvalho A.M., Santos-Buelga C., Fernandes I.P., Barreiro M.F., Ferreira I.C. (2014). Exploring the antioxidant potential of *Helichrysum stoechas* (L.) Moench phenolic compounds for cosmetic applications: Chemical characterization, microencapsulation and incorporation into a moisturizer. Ind. Crop. Prod..

[37] Martins A., Barros L., Carvalho A.M., Santos-Buelga C., Fernandes I.P., Barreiro F., Ferreira I.C.F.R. (2014). Phenolic extracts of *Rubus ulmifolius* Schott flowers: Characterization, microencapsulation and incorporation into yogurts as nutraceutical sources. Food Funct..

[38] Singh B., Singh J.P., Kaur A., Singh N. (2017). Phenolic composition and antioxidant potential of grain legume seeds: A review. Food Res. Int..

[39] Miano A.C., Augusto P.E.D. (2018). The Hydration of Grains: A Critical Review from Description of Phenomena to Process Improvements. Compr. Rev. Food Sci. Food Saf..

[40] Wiesinger J.A., Cichy K.A., Glahn R.P., Grusak M.A., Brick M.A., Thompson H.J., Tako E. (2016). Demonstrating a Nutritional Advantage to the Fast-Cooking Dry Bean (*Phaseolus vulgaris* L.). J. Agric. Food Chem..

